# Fall-related injury among patients with vestibular schwannoma

**DOI:** 10.1371/journal.pone.0304184

**Published:** 2024-06-14

**Authors:** Christine Ölander, Maria Feychting, Per Olof Eriksson, Göran Laurell, Mats Talbäck, Stina Ek

**Affiliations:** 1 Department of Surgical Sciences, Section of Otolaryngology and Head Neck Surgery, Uppsala University, Uppsala, Sweden; 2 Unit of Epidemiology, Institute of Environmental Medicine, Karolinska Institute, Stockholm, Sweden; Yonsei University Wonju College of Medicine, REPUBLIC OF KOREA

## Abstract

Vestibular schwannoma can cause vestibular dysfunction; however, conflicting evidence exists regarding whether this affects the incidence of fall-related injuries in this patient population. This matched cross-sectional and cohort study assess the risk of fall-related injuries in patients with vestibular schwannoma. The study included patients with vestibular schwannoma treated at a tertiary referral hospital in Sweden between 1988 and 2014. Information on fall-related injuries was obtained from the National Patient Register, and matched population comparisons were randomly selected in a 1:25 ratio. Fall-related injuries occurring pre- (within 5 years before the diagnosis of vestibular schwannoma) and post-diagnostically (up to 3 years after diagnosis or intervention) were registered. The association between vestibular schwannoma and fall-related injuries was estimated using logistic regression and Cox proportional hazards analyses. We identified 1153 patients with vestibular schwannoma (569 [49%] women and 584 [51%] men), and 28815 population comparisons. Among the patients, 9% and 7% had pre- and post-diagnostic fall-related injuries, respectively, and among the comparisons, 8% and 6% had pre- and post-diagnostic fall-related injuries, respectively. There was no increased risk of pre- (OR 1.14; CI 0.92–1.41) or post-diagnostic 1 year (HR 1.16; CI 0.87–1.54) or 3 years (HR 1.11; CI 0.89–1.29) fall-related injury among the total patient cohort. In age-stratified analyses, we found an increased risk of pre-diagnostic fall-related injury among patients aged 50–69 years (OR 1.42; CI 1.10–1.88). Patients who underwent middle fossa surgery, regardless of age, had an increased risk of post-surgery fall-related injury within 3 years of follow-up (HR 2.68; CI 1.06–6.81). We conclude that patients with vestibular schwannoma have a low risk of enduring fall-related injuries. Middle-aged patients with dizziness and fall-related injuries should be considered for a vestibular clinical evaluation. Our results highlight the importance of rehabilitation in avoiding future fall-related injuries among patients undergoing middle fossa surgery.

## Introduction

Vestibular schwannoma (VS) is a benign, slow-growing tumor originating from the Schwann cells surrounding the vestibular branch of the eight cranial nerve [1]. The current primary approach of managing VS includes serial magnetic resonance imaging (MRI) scans over time (i.e., “wait and scan”). If the tumor grows or when neurological complications occur, surgical interventions (e.g., microsurgery (MS) or radiosurgery) aimed at preventing debilitating sequelae and maintaining quality of life may be needed [1]. The choice of intervention depends on several factors, such as tumor location and size, comorbidity conditions and whether hearing preservation is a major goal [2]. When using the trans labyrinthine (TL) approach, hearing and vestibular functions are lost postoperatively [3], in contrast to middle fossa (MF) or retro-sigmoidal (RS) approach, which aims to preserve hearing [4, 5]. The objective of Gamma-Knife Stereotactic Radiosurgery (GK-SRS) is to arrest the tumor growth while minimizing the risk of harm to adjacent structures [6].

When diagnosed, the majority of patients with VS present with unilateral hearing loss and tinnitus, as well as considerably varying vestibular function [7–10] and dizziness [11, 12]. The latter is a risk factor of traumatic falls [13], and it is not known how long before being diagnosed with VS, the symptoms occur.

VS is commonly slow growing [1], which enables a gradual central adaptive compensation for progressive vestibular function reduction [7, 14, 15] and minimizes the VS-related vertigo symptoms [16]. Moreover, there is a discrepancy between the measured vestibular impairment and perceived dizziness disability [17]. That is, owing to the degree of central compensation, the symptoms of vertigo can be mild or even non-existent, despite a profound loss of vestibular function.

Increased numbers of VS cases have been diagnosed in the last few decades, particularly among older individuals [1, 18, 19]. Aging is also strongly associated with the risk of fall-related injury [13, 20, 21]. A limited number of studies have reported the incidence of self-reported falls among patients with VS [22, 23]. However, to our knowledge, the incidence of objectively registered fall-related injuries among patients with VS, including the association with different interventions, remains unknown.

The primary aim of this study was to investigate the risk of fall-related injuries among patients with VS both before diagnosis and thereafter by using objective data from national population register. The secondary aim was to investigate any differences in the fall-related injuries with respect to the different means of intervention.

## Methods

This matched, cross-sectional study of pre-diagnostic, and cohort study of post-diagnostic fall related injuries was registry-based and included patients diagnosed with or treated for VS at Uppsala University Hospital, Sweden, between 01-01-1988 and 12-31-2014. A cohort for comparison (i.e., a VS-free population) from the same geographical area was identified using the Total Population Register (TPR) [24]. Through personal identification numbers (PIN) unique to each Swedish resident, patients with VS and comparisons were investigated using the National Health Data and administrative registers.

This study was approved by the Swedish ethical review authorities (2011/634–31/4, 2016/27–32, 2019–05415, 2021–04376, 2021–04575). After ethical approval 2019, the data in the clinical register were compiled according to the study design. During this period the researchers had access to patients’ personal information. In December 2021 the compiled data was sent to Statistics Sweden for linkage with registers and pseudonymization. During the analysis of the data, the researchers had no access to personal information. Informed consent was waived due to the large sample size and use of pseudonymized register data, and the retrospective nature of the study. This study adhered to the Strengthening the Reporting of Observational Studies in Epidemiology reporting guidelines.

### Comparison of patients with VS and VS-free population

A total of 1344 patients diagnosed with unilateral VS were identified in the registry. Medical records were reviewed to determine the date of diagnosis (i.e., date for the first radiological report demonstrating a sporadic VS), referred to as the “index date”.

Patients with missing records regarding the index date (n = 166) or neurofibromatosis type 2 diagnosis (n = 25) were excluded. If an intervention was performed, type and date were recorded. VS was histopathologically confirmed in all the patients with MS.

Using the TPR, we randomly selected 25 controls free of VS per patient with VS, matched for age, sex, and geographic region of residence on the date of diagnosis of the patient with VS. As some regions had fewer inhabitants, a couple of patients with VS did not have 25 matches but slightly fewer than that (in total, a difference of 10 missing comparisons). By matching on date of diagnosis and age, we control for the changes in population composition, health care structure, and diagnosis precision that have appeared over the study period.

The VS-cohort was distributed to “wait-and-scan,” and different intervention alternatives of MS (TL, MF, or RS) or GK-SRS. Time between diagnosis and MS or GK-SRS intervention differed substantially (median [interquartile range (IQR)], 159 [475] days) and all patients were categorized as “wait-and-scan” until the date of intervention. Due to the small number of patients treated with the RS approach (n = 4), they were grouped with patients using the MF approach in the statistical analysis. The main rationale for this grouping was that both the methods aimed to preserve hearing.

### Outcomes

“Fall-related injury” was defined as a fall caused by low-energy trauma, without the involvement of other persons, resulting in hospitalization or receipt of outpatient specialist care because of the fall [25]. Diagnoses from the International Classification of Disease, 9^th^ and 10^th^ revisions (ICD 9 and ICD 10) were used to identify the types of falls and injuries. The included external cause codes were: ICD-9: E880, E885, E887, E888; ICD-10: W00, W01, W5-W10; W17-W19. Fall-related injuries were identified from the national patient register (NPR), with national coverage of hospitalizations since 1987 and outpatient specialist care since 2001 [26]. The NPR is an administrative registry with a nearly full national coverage from start up until today [26], and for this project we had information from the NPR until December 31^st^, 2015.

Fall-related injuries up to 5 years prior to the index date and up to 3 years after the index date were recorded. Each participant contributed to the fall-related injuries (yes or no) in both the pre- and post-diagnostic periods. This was regardless of the number of care visits due to a fall or if the participant endured more than one fall-related injury.

### Covariates

The comorbidities were classified according to the Charlson Comorbidity Index (CCI) [27]. CCI were extracted from the NPR through ICD codes from hospitalizations/specialist care visits during the 5 years prior to the index date and thereafter categorized into three different groups: 0, 1, and 2+. The cohabitation status was identified from the integrated database for the Labor Market Research register within 1 year prior to the index date [28] and categorized as living with someone or living alone.

Information about the vital status of the participants was obtained from the National Cause of Death Registry [29].

### Statistical analysis

Descriptive statistics were used to summarize the sociodemographic and medical characteristics. The number of individuals diagnosed with VS at Uppsala University Hospital over the study period was shown graphically. A descriptive graph was created to present the healthcare burden due to fall-related injuries, allowing for more than one fall during the study period per individual during the main study period. The graph presents the presence of fall-related injuries (yes/no) per 90-day period during the 5 years prior to the index date and up to 1 year after, separately for patients with VS and comparisons.

In a cross-sectional analysis, we assessed the risk of any fall-related injury during the 5 years prior to VS diagnosis using logistic regression models to estimate the odds ratios (OR) and 95% confidence intervals (CI). The models were adjusted for matching factors (age, sex, and region) in Model 1 and for cohabitation status and CCI in Model 2. In addition, because both VS diagnosis and fall-related injury are associated with age, we stratified the patients by age groups into <50, 50–69, and 70+ years.

To assess the short- and long-term risk of fall-related injury after the index date, we followed-up the patients with VS during 1 and 3 years, respectively. Hazard ratios (HR) and 95% CI were calculated using the Cox proportional hazards model. Each participant contributed person-time until a fall-related injury, intervention procedure (MS or GK-SRS), emigration, date of death, or end of follow-up (12-31-2015), whichever occurred first.

To investigate the impact of the intervention for VS (MS or GK-SRS) on fall risk, we additionally performed Cox proportional hazards analyses in the subgroup of the study population that underwent MS or GK-SRS starting on the day of intervention instead of the index date (n = 655) (43 patients with VS and their matched comparisons were not included because the date of treatment was after the study period). Previously described confounders and stratifications were used.

To investigate the possible impact of the increased number of patients diagnosed with VS over the study period, we also performed the above mentioned analyses stratified by diagnosis year intervals (1988–2000, 2001–2010 and 2011–2014).

All statistical analyses were performed using Stata version 16 (StataCorp, College Station, TX, USA).

## Results

In total, we identified 1153 patients with VS and 28,815 matched VS-free population comparisons, who were included in the study. The median (IQR) age at VS diagnosis was 56 (19) years, and 569 (49%) patients were women. The number of individuals that were diagnosed with VS increased over the study period (S1 Fig). Among the patients with VS, 452 (39%) were considered for continued observation “wait-and-scan,” 614 (53%) underwent MS (528 (46%) cases with TL approach, 82 (7%) cases with MF approach, and 4 (0.5%) cases with RS approach and 87 (8%) cases were treated with GK-SRS. The characteristics of the study participants are presented in Table 1.

**Table 1:** Characteristics of the study population by age groups.

	Vestibular schwannoma patients				Comparisons			
Age groups	All	<50	50–69	70+	All	<50	50–69	70+
n	1153	355	615	183	28815	8836	15370	4609
**Age, median (IQR)**	56,0 (19,2)	40,2 (10,8)	58,5 (9,4)	73,1 (6,3)	55,9 (19,2)	40,2 (10,4)	58,4 (9,5)	73,1 (6,3)
**Women, n (%)**	569 (49,4)	169 (47,6)	301 (48,9)	99 (54,1)	14218 (49,3)	4247 (48,1)	7472 (48,6)	2499 (54,2)
**Men, n (%)**	584 (50,7)	186 (52,4)	314 (51,1)	84 (45,9)	14597 (50,7)	4589 (51,9)	7898 (51,4)	2110 (45,8)
**Treatment, n (%)**								
Wait and scan	452 (39,2)	69 (19,4)	264 (42,9)	19 (65,0)	NA	NA	NA	NA
Translabyrinthine surgery	528 (45,8)	224 (63,1)	254 (41,3)	50 (27,3)	NA	NA	NA	NA
Middle fossa surgery^a^	86 (7,5)	47 (13,2)	39 (6,3)	0	NA	NA	NA	NA
Gammaknife radiation	87 (7,5)	15 (4,2)	58 (9,4)	14 (7,7)	NA	NA	NA	NA
**Cohabitation status, n (%)**								
Living alone	475 (41,2)	199 (56,1)	218 (35,5)	58 (31,7)	13430 (46,6)	5209 (58,9)	6175 (40,2)	2049 (44,5)
Living with someone	678 (58,8)	156 (43,9)	397 (64,6)	125 (68,3)	15385 (53,4)	3630 (41,1)	9195 (59,8)	2560 (55,5)
**Charlson comorbidity index, n. (%)**								
0	1001 (86,8)	335 (94,4)	533 (86,7)	133 (72,7)	24996 (86,8)	8473 (95,9)	13406 (87,2)	3117 (67,6)
1	68 (5,9)	10 (2,8)	37 (6,0)	21 (11,5)	1758 (6,10)	231 (2,6)	941 (6,1)	586 (12,7)
2+	84 (7,3)	10 (2,8)	45 (7,3)	29 (15,9)	2061 (7,2)	132 (1,5)	1023 (6,7)	906 (19,7)
**1-year mortality**	4	1	2	1	272	8	110	154

^a^Middle fossa approach including four patients operated with retro sigmoidal approach.

**Fig 1:**
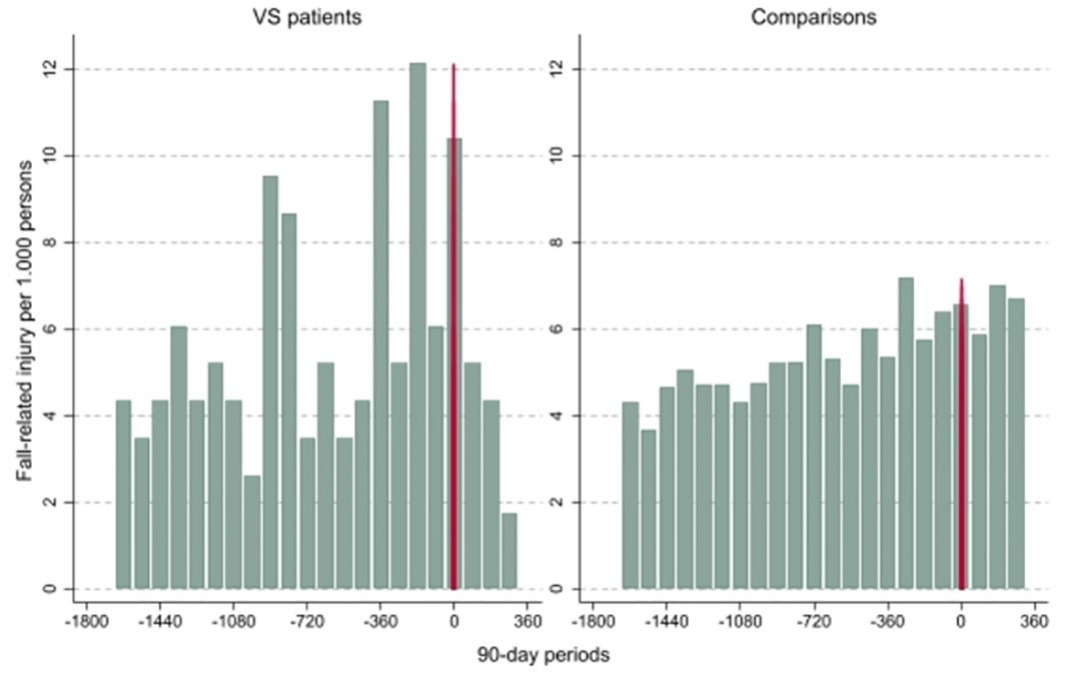
Any fall-related injury/1000 persons per 90-day period 5 years before and 1 year after the index date by patients with VS and comparisons.

Fig 1 shows at least one fall-related injury (yes/no) per 90-days/1000 individuals stratified by patients with VS and comparisons 5 years before and 1 year after the index date. The plot shows a visual tendency towards an increased number of registered fall-related injuries among patients with VS prior to the index date, but not after.

In recent decades, the incidence of VS has steadily increased globally [30]. Stratified analysis to investigate whether time of diagnosis had an influence on the risk of falls, did not present any changes regarding fall-related injury (S1 Table).

### Risk of fall-related injury before index date

**Fig 2:**
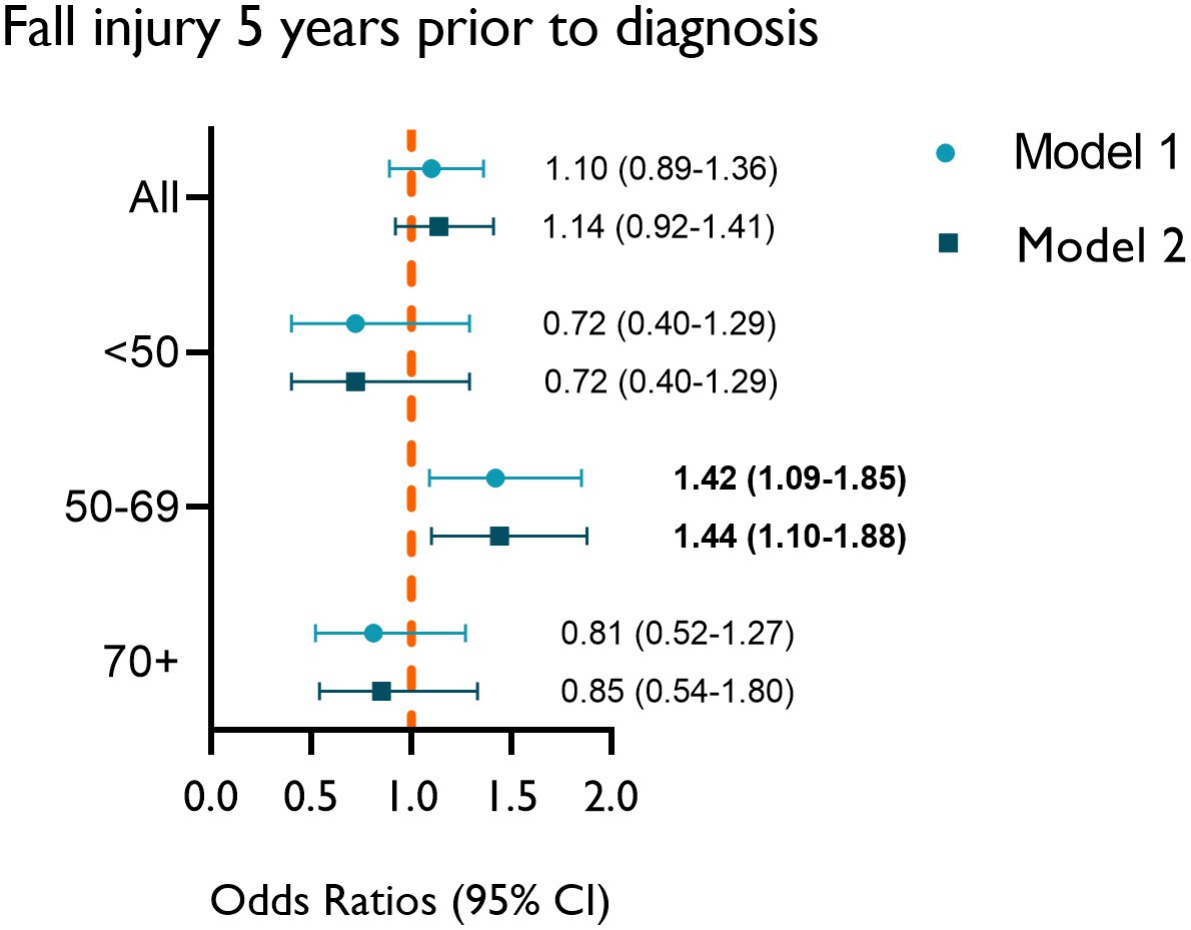
Fall-related injury prior to diagnosis. Risk of having one or more fall-related injury 5 years prior to VS diagnosis, all patients and according to age groups. Model 1: adjusted for age, sex, and region; Model 2: additionally adjusted for cohabitation and CCI (patients with VS, n = 1153; comparisons, n = 28815).

During the 5 years period before the index date, 101 (9%) patients with VS and 2313 (8%) comparisons were hospitalized or received outpatient specialist care because of a fall-related injury at least once. There was no increased risk of fall-related injury among the total VS cohort (OR 1.14; CI 0.92–1.41) after adjusting for age, sex, region, cohabitation status, and CCI. Stratification by age groups revealed an increased risk for fall-related injury among patients with VS between the ages of 50–69 years (OR 1.42; CI 1.10–1.88; n = 615) in the fully adjusted model, while the youngest and oldest age groups did not show any significant difference between patients with VS and comparisons (Fig 2).

### Risk of fall-related injury after index date

During the year after the index date, a total of 50 (4%) patients with VS and 1092 (4%) comparisons were hospitalized or received outpatient specialist care for fall-related injuries. During the 3 years follow-up period, the number of patients with fall-related injuries increased to 80 (7%) patients with VS and 1813 (6%) comparisons. There was no increased risk of fall-related injuries among patients with VS neither up to 1 year (HR 1.16; CI 0.87–1.54) nor 3 years (HR 1.11; CI0.89–1.29) after the index date in the fully adjusted model. None of the age groups showed an increased risk (Table 2).

**Table 2:** Risk of a fall-related injury, 1 and 3 years after a) diagnosis, and b) active intervention, presented as hazard ratios (HR) and the corresponding 95% CI. Model 1: adjusted for age, sex, and region; Model 2: additionally adjusted for cohabitation and CCI.

	Hazard ratios (95% CI)			
	1 year		3 years	
	Model 1	Model 2	Model 1	Model 2
**From date of diagnosis, VS patients n = 1150; comparisons n = 28715**				
All	1.14 (0.86–1.52)	1.16 (0.87–1.54)	1.09 (0.87–1.37)	1.11 (0.89–1.29)
<50 years	1.44 (0.86–2.43)	1.46 (0.87–2.46)	1.15 (0.69–1.89)	1.15 (0.70–1.90)
50–69 years	1.11 (0.74–1.65)	1.11 (0.74–1.65)	1.17 (0.86–1.58)	1.17 (0.86–1.58)
70+ years	0.93 (0.50–1.75)	0.96 (0.51–1.8)	0.92 (0.59–1.44)	0.95 (0.61–1.49)
**From date of active intervention, VS patients n = 655; comparisons n = 16346**				
All	0.73 (0.32–1.64)	0.75 (0.33–1.68)	1.31 (0.90–1.90)	1.33 (0.92–1.93)
<50 years	0.44 (0.06–3.21)	0.45 (0.06–3.24)	1.32 (0.65–2.68)	1.33 (0.65–2.71)
50–69 years	1.22 (0.50–3.00)	1.24 (0.51–3.05)	1.47 (0.89–2.43)	1.49 (0.90–2.47)
70+ years	^a^	^a^	0.97 (0.40–2.39)	1.02 (0.42–2.50)
**Stratified by different active interventions**				
Translabyrinthine	0.61 (0.23–1.65)	0.63 (0.24–1.71)	1.15 (0.74–1.79)	1.19 (0.77–1.84)
Middle fossa^b^	2.79 (0.65–12.01)	2.59 (0.58–11.50)	**2.63 (1.05–6.60)**	**2.68 (1.06–6.81)**
GK-SRS^c^	^a^	^a^	1.36 (0.43–4.36)	1.33 (0.42–4.26)

^a^Insufficient power

^b^Middle fossa approach included four patients who underwent a sigmoidal approach

^c^gamma knife stereotactic radiosurgery

### Risk of fall-related injuries after intervention

On studying the risk of fall-related injuries in the group that received intervention (MS or GK-SRS) and their comparisons, we found no increased risk over time of fall-related injuries, neither with a follow-up of 1 year nor 3 years after the date of intervention (1 year: HR 0.75, CI 0.33–1.69; 3 years: HR 1.34, CI 0.92–1.94) (Table 2).

To evaluate whether the type of active VS intervention affected the risk of fall-related injuries, a subgroup analysis was performed for each intervention. An increased risk was only seen for a 3-year follow-up among patients with VS who had undergone an MF or RS surgery (HR 2.68; CI 1.06–6.81 in the fully adjusted model) (Table 2). The four patients who underwent RS surgery had no registered fall-related injuries after the intervention. However, the analyses stratified by intervention had low power, and analyses for the GK-SRS over a year of follow-up could not be performed at all.

## Discussion

In this matched cross-sectional cohort study, no increased risk of fall-related injury was found among patients with VS. In general, the number of patients suffering from fall-related injuries was low, and the incidence was similar between the groups. However, patients with VS aged 50–69 years showed an increased risk of fall-related injury up to 5 years prior to the index date compared with their comparisons. We also found an increased risk of postoperative fall-related injuries among patients with VS who underwent MF surgery.

Accurate and sufficient data are pivotal for the monitoring and comparison of fall-related injuries among patients and comparisons. According to the ICD 9 and ICD 10, the coding is not unique to VS but includes all benign neoplasms of the cranial nerves (225.1 and D33.3). Therefore, a clinical registry of patients with a proven VS diagnosis is crucial. Linking our large VS cohort with full-coverage national registers [31] provided us with a unique opportunity to investigate the association between VS diagnoses and objective fall injuries. The ability to assess the incidence of fall-related injuries using reliable data provides valuable information and useful insights into the consequences of sporadic VS.

The data from earlier studies were generally based on self-reported falls. Huang et al. reported the proportion of self-reported falls among 30 patients to be 10%. After adjusting for risk factors such as age, sex, and CCI, no significant increase in self-reported falls was found compared to patients with other vestibular diagnosis [22]. Rigby et al. reported self-reported falls in 130 patients with VS undergoing microsurgery; 24 and 23% of the patients reported falls pre- and postoperatively, respectively [23]. In this study, 9% of the patients with VS had fall-related injuries before diagnosis, and 7% had injuries post-diagnosis. Saman et al. presented 63 patients with untreated VS. Based on the results of the functional gait assessment score, the authors predicted a higher risk of falling in patients older than 60 years [32]. This is in contrast to our study, in which we did not find an increased risk of fall injuries among patients with VS aged over 69 years.

Most patients with VS have vestibular deficits in the tumor-affected ear [7], and numerous studies have presented the risk of fall-related injury among patients with asymmetric vestibular function originating from other vestibular disorders [33–36]. According to Donovan et al., more than one in two patients who suffered falls had objective signs of vestibular deficit [37]. Baldursdottir et al. reported an increased prevalence of vestibular deficits in patients aged 50–75 years with fall-related wrist fractures [38]. The slow growth of VS might affect the vestibular function and central compensation in a different way compared with vestibular deficits in patients with more acute vestibular disorders and unpredictable attacks of vertigo [32]. In this study we do not have access to objective data regarding vestibular function, and the results shall be interpreted as investigating association between fall injuries and VS, not causality with vestibular function. In accordance with the studies presented above, one can still speculate whether vestibular hypofunction contributed to the results.

Age is a risk factor for falling [20] and is reported to affect 38% of people over the age of 65 years [39]. In our analyses, we adjusted for age, sex, CCI, and cohabitation status, all of which are known to be strong risk factors for falls, especially at an older age. The results showed that patients aged 50–69 years had an increased risk of fall-related injuries before VS diagnosis. It is possible that younger patients can compensate for the balance impairment, while the older population has accumulated several other risk factors for falls (such as comorbidity, impaired physical function, and cognitive function), with a greater impact than the symptoms from VS. Even though the age range is wide, the findings suggest that patients aged 50–69 years are most affected by VS prior to diagnosis. In addition, this group would probably benefit from an early detection of VS from the perspective of a fall prevention. After diagnosis, none of the three age groups with VS had a higher risk of fall-related injuries. Awareness of the disease and its associated risks may affect the lifestyle and behavior of patients.

By studying the different treatment interventions, we found an increased risk of fall-related injuries among patients with VS undergoing MF surgery. Patients who underwent MF surgery were younger and had smaller tumors. According to the results presented by Batuecas-Caletrio et al., patients with VS and severe vestibular loss in the tumor-affected ear before microsurgery compensate faster after surgery [40, 41]. Although it has been reported that the tumor size does not influence the degree of vestibular function [42], one can hypothesize that patients subjected to MF surgery due to smaller tumors have preoperatively better vestibular function and postoperatively reduced central compensation. Therefore, these patients might postoperatively suffer from more acute vestibular symptoms and may be at a higher risk of fall-related injuries after surgery. However, the increased risk of fall related injury after MCF surgery should be interpreted with caution, given the low number of patients subjected to MCF surgery in our cohort.

The major strength of this study was the large VS cohort combined with reliable national register data of fall injuries, as compared to self-reported falls. Owing to the linkage to the PIN, the loss of follow-up data was minimal [43]. From the NPR, we identified fall-related injuries that were severe enough to prompt the patient to seek medical attention. However, most falls do not result in injuries [44], and consequently we may have underestimated our association. However, the more severe falls are likely to have stronger negative impact on the individual. Also, our linkage to high quality registers limit potential recall biases, a known issue with self-reported falls.

Despite the large VS cohort and close to full coverage of healthcare registers, the number of registered fall-related injuries was low. The low incidence of fall-related injuries affected the power of the statistical tests. In addition, the low incidence of falls made it difficult to compare the type of fall injury and injury severity between patients with VS. It was also difficult to separate several healthcare visits owing to a single and different fall-related injuries. To compensate for this, we plotted any fall-related injury/1000 individuals among both patients with VS and comparisons in 90-day intervals. We did not find any statistical difference between patients with VS and comparisons; however, there was a tendency for more healthcare visits due to fall-related injuries among patients with VS in the years prior to diagnosis. It is possible that a pathological falling pattern can make both the patient and the clinician suspect a dizziness problem, thereby initiating an accurate clinical examination leading to a diagnosis of VS.

A strong relationship exists between the degree of dizziness and quality of life among patients with VS [45–47]. In our study, individual symptoms, such as the degree of dizziness or limitations in daily life, were elusive factors that can be important in future studies. Also, other comorbidities known to increase risk of falls (e.g., neurological deficits or ortho related) could be of importance.

## Conclusion

To our knowledge, this is the first study to present the risk of fall-related injuries among patients with VS compared with VS-free comparisons using objective data from national population register. In conclusion, the risk of fall-related injury in patients with VS is low. However, we found an increased risk of fall-related injuries among middle-aged patients before being diagnosed with VS and postoperatively in patients after MF surgery. This highlights the importance of the vestibular clinical evaluation among middle-aged patients suffering from dizziness and fall-related injuries, as well as rehabilitation of patients with VS treated with MF surgery to avoid fall injuries.

## Supporting information

S1 Fig Number of patients diagnosed with VS per year. Number of patients diagnosed with VS at Uppsala University Hospital during the timespan 1988–2014.
(PDF)

S1 Table Risk of fall-related injury depending on time of diagnosis. Risk of fall-related injury depending on when during the study period the patient is diagnosed with VS (1988–2000; 2001–2010; 2011–2014).
(PDF)
