# Supplementary material for: Fall-related injury among patients with vestibular schwannoma
Source: PLoS One. 2024 Jun 14;19(6):e0304184. doi: 10.1371/journal.pone.0304184 (PMC11178211; doi:10.1371/journal.pone.0304184)
Supplement: S1 Fig — Number of patients diagnosed with VS at Uppsala University Hospital during the timespan 1988–2014. (PDF) [file pone.0304184.s001.pdf]

Supporting information – S1 Fig

Number of patients diagnosed with VS per year

Number of patients diagnosed with VS during the timespan 1988 – 2014 is presented in S1 Fig.

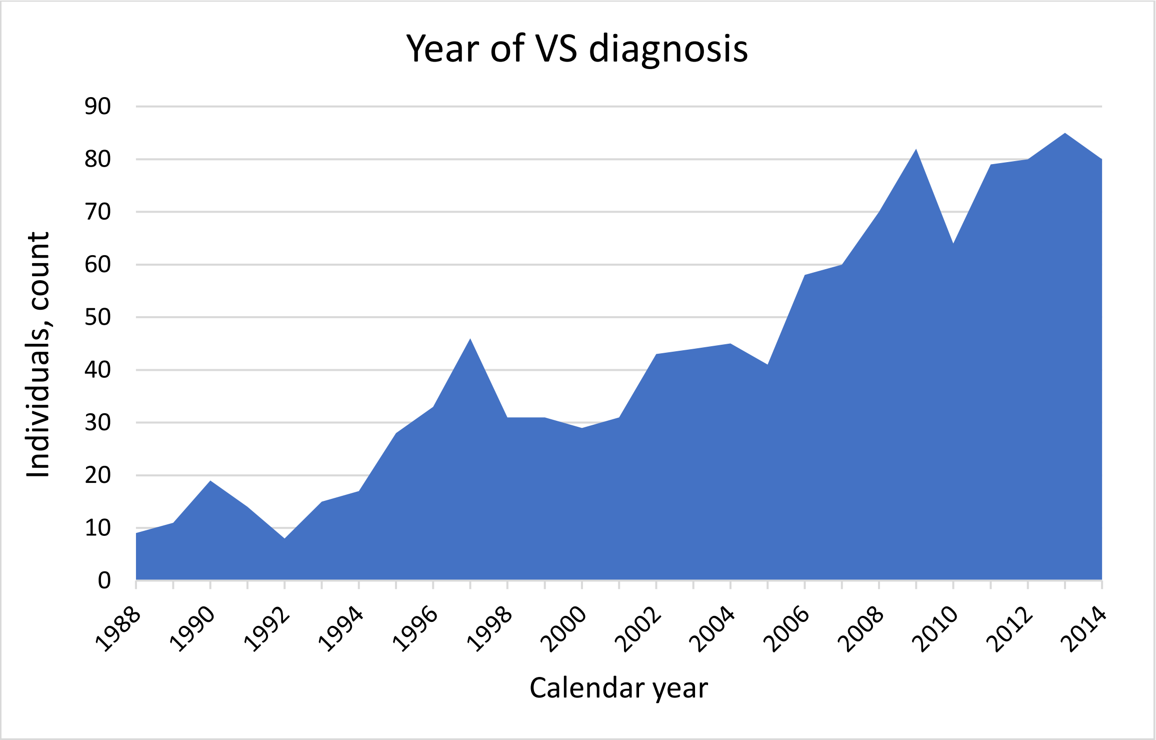

S1 Fig. Graphic presentation of number of patients diagnosed with VS per year.
