# Supplementary material for: Fall-related injury among patients with vestibular schwannoma
Source: PLoS One. 2024 Jun 14;19(6):e0304184. doi: 10.1371/journal.pone.0304184 (PMC11178211; doi:10.1371/journal.pone.0304184)
Supplement: S1 Table — Risk of fall-related injury depending on when during the study period the patient is diagnosed with VS (1988–2000; 2001–2010; 2011–2014). (PDF) [file pone.0304184.s002.pdf]

Supporting information - S1 Table

Risk of fall-related injury depending on time of diagnosis

Risk of fall-related injury depending on when during the study period the patient is diagnosed with VS is presented in table SI-1B. The data is adjusted for age, sex, and region (model 1); and additionally for cohabitation and CCI (model 2)

S1 Table Risk of fall-related injury depending on time of VS diagnosis.

Year of diagnosis	1988-2000	2001-2010	2011-2014
n	7355	13 982	8423
**Up to five years prior to diagnosis**[a]			
Model 1	1.41 (0.65–3.06)	1.12 (0.83–1.50)	1.04 (0.74–1.46)
Model 2	1.50 (0.69–3.25)	1.14 (0.85–1.53)	1.08 (0.77–1.51)
**After diagnosis – follow up one year**[b]			
Model 1	1.02 (0.49–2.17)	1.26 (0.89–1.78)	0.94 (0.50–1.78)
Model 2	1.02 (0.48–2.17)	1.26 (0.89–1.79)	0.97 (0.51–1.82)
**After diagnosis – follow up three year**[b]			
Model 1	1.08 (0.55–2.10)	1.19 (0.90–1.57)	0.89 (0.56–1.42)
Model 2	1.07 (0.55–2.09)	1.20 (0.91–1.58)	0.91 (0.57–1.46)

[a]Risk of fall-related injury five years prior to VS diagnosis presented as Odds Ratio (OR) and 95% confidence intervals (CI)

[b]Risk of fall-related injury after diagnosis (one and three years respectively) presented as Hazard ratios (HR) and 95% CI.
